# Can Hydrographic Data Provide Evidence That the Rate of Oceanic Uptake of Anthropogenic CO_2_ Is Increasing?

**DOI:** 10.1371/journal.pone.0071920

**Published:** 2013-08-20

**Authors:** William Carlisle Thacker

**Affiliations:** University of Miami and Atlantic Oceanographic and Meteorological Laboratory, Miami, Florida, United States of America; University of Vigo, Spain

## Abstract

Predictions of the rate of accumulation of anthropogenic carbon dioxide in the Pacific Ocean near 32°S and 150°W based on the P16 surveys of 1991 and 2005 and on the P06 surveys of 1992 and 2003 underestimate the amount found in the P06 survey of 2009–2010, suggesting an increasing uptake rate. Assuming the accumulation rate to be constant over the two decades, analyses using all five surveys lead to upward revision of the rates based only on the first four. On the other hand, accumulation rates estimated for 2003–2010 are significantly greater than those for 1991–2003, again suggesting an increasing uptake rate. In addressing this question it is important to acknowledge the limitations of the repeat hydrography and consequent uncertainties of estimated accumulation rates.

## Introduction

A recent paper [1] introduced a two-regression method for estimating the rate of accumulation of anthropogenic carbon dioxide within a limited region of the ocean. The first regression serves as a filter, removing the variability of the observed total inorganic carbon that should be present in the absence of anthropogenic sources while leaving the anthropogenic signal in the residuals together with unexplained variability that might be regarded as noise. The second regression fits a linear function of time to the residuals to account for a systematic temporal trend that can be identified as the anthropogenic signal, with the slope of the trend line providing an estimate of the accumulation rate. This method was illustrated using data obtained from repeated surveys along 32.5°S (P06) and along 150°W (P16) in the South Pacific within the period from 1991 to 2005. The rates of accumulation were estimated for the region surrounding the intersection of the survey tracks for two layers, one centering on 200 dbar and the other on 400 dbar. For each layer, six different sets of regressors were used to account for the natural variability in the region and accumulation rates were estimated from each model’s residuals.

With no accumulation before the industrial revolution, the rate had to have increased from zero, and it is reasonable to expect that it is still increasing. The simplest argument is that the increasing amounts of atmospheric CO_2_ provide more for the ocean to take up. The observation-based fact that carbon appears to be increasing faster in the 200 dbar layer than in the deeper 400 dbar layer [1] also supports the notion of accelerating accumulation, as the anthropogenic signal takes longer to reach the deeper layer. The objective of this study is to see whether the addition of the more recent data from 2009–2010 allows for observational evidence of accelerating rates within the separate layers.

The new data are found to have a greater amount of anthropogenic carbon than would be expected given the accumulation rates inferred from the four previous surveys. While the excess is relatively small and within the range of unexplained variability, the fact that it is seen consistently for all models used to filter out the natural environmental variability might be taken as an indication of an increasing rate of accumulation of anthropogenic carbon. Similarly, revised analyses of the data from all five surveys, regardless of the filtering, lead to upwardly revised accumulation rates. And rates estimated using data from the first through third surveys and separately using data from the third through fifth surveys indicate substantial increases regardless of how natural environmental variability is treated.

## Data

**Figure 1:**
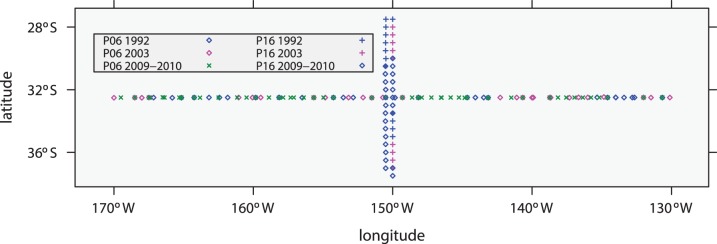
Locations of stations contributing data to this study.

**Figure 2:**
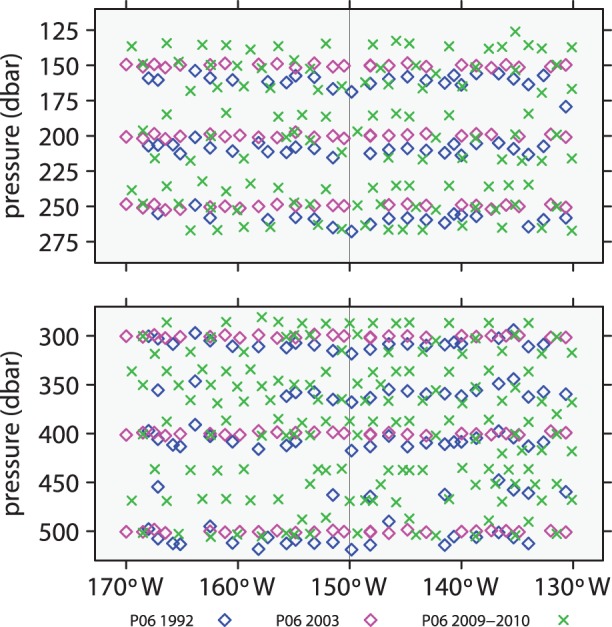
Locations of data from P06 surveys. The vertical lines indicates where P06 and P16 cross. Blue indicates data from 1992; magenta, 2003; green, 2009 and 2010.

**Figure 3:**
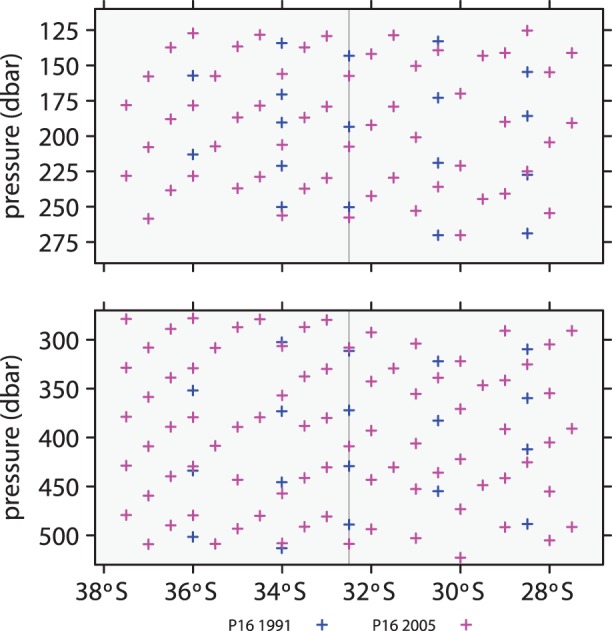
Locations of data from P16 surveys. The vertical lines indicates where P06 and P16 cross. Blue indicates data from 1991; magenta, 2005.

The data considered here, which are freely available from CLIVAR, are subsets of P06 and P16 surveys that were obtained in the vicinity of the intersection of the two survey lines as illustrated in figure 1. Because evidence of accumulating anthropogenic CO_2_ is expected to be strongest in the upper ocean, the study focuses on data within two upper-ocean layers, 125–275 dbar and 275–525 dbar, as illustrated in figures 2 and 3. The shallower layer has a stronger anthropogenic signal but greater variability due to near-surface processes, while the deeper layer has a smaller signal and less environmental noise. If the signal-to-noise ratio is large enough, there is a possibility of detecting a changing rate of accumulation of anthropogenic carbon.

The number of samples available to this study, which are listed in Table 1, varies from survey to survey with the 1991 P16 survey providing fewest data and the 2009–2010 P06 survey providing the most. Consequently, variability over latitude is more poorly sampled than variability over longitude. Descriptions of the data can be found in [2]–[7].

**Table 1:** Number of samples in study region.

	200 dbar	400 dbar
1991 P16	18	18
**1992 P06**	**68**	**94**
**2003 P06**	**74**	**77**
2005 P16	57	83
**2009–10 P06**	**115**	**154**

Local averages of the observed amounts of total inorganic carbon for each survey provide a time series that might serve to indicate an increase in the region. While such volume averages are clearly more stable than the individual point observations, as they serve to remove some of the natural variability that might be confounded with an anthropogenic signal, the limited nature of the sampling brings into question how representative of the natural variability of the region over the two decades of this study is the variability seen at the time of the surveys. This argues for combining data from multiple surveys so that the analysis might be based on as much of the variability as possible.

By working with the surveys collectively it is possible to exploit the co-variability of inorganic carbon with other measured variables in an attempt to identify and to remove the consequences of horizontal and vertical shifts in the water mass properties and of mixing process in the upper ocean, so that remaining systematic temporal changes might be attributed to anthropogenic causes alone. Although co-variability of carbon with other environmental variables can mitigate this concern, there is still the issue of whether the sample is sufficient to capture this co-variability. As these are the only data available, it is necessary to proceed while recognizing their limitations.

## Models

The models listed in Table 2 provide alternative characterizations of the natural variability of dissolved inorganic carbon through its co-variability with the regressors. They are exactly the same models as those discussed when the two-regression method was introduced [1]. The default model m0, which has only the intercept term, makes no attempt to account for non-anthropogenic variability other than through its averaging of the carbon observations. Because near-surface process stratify water according to its density, all of the other models have potential density as a regressor. In addition model m2, m3, m4, and m5 have one or more additional regressors to account for variability due to biogeochemical processes. These models are fitted separately to the data in the 200 dbar layer and to those in the 400 dbar layer, as the two layers experience different variability.

**Table 2:** Regressors for the models used to account for environmental variability are indicated by crosses.

	1	density	nitrate	silicate	aou
m0	x				
m1	x	x			
m2	x	x	x		
m3	x	x	x	x	
m4	x	x	x		x
m5	x	x	x	x	x

The intercept term is indicated by 1, apparent oxygen utilization by aou.

After being fitted to the data from the first four surveys, each of these models characterizes the non-anthropogenic part of the variability of total dissolved inorganic carbon as being linearly proportional to the variability of the regressors. Each model’s residuals contain its version of the anthropogenic signal plus noise. Because the intercept terms contain the regional average of inorganic carbon from these four surveys (2077.3 for 200 dbar and 2122.4 for 400 dbar), that average is missing from the residuals. If the regression is to be regarded as filtering out variability without removing the mean, the means should be added back to the residuals.

**Figure 4:**
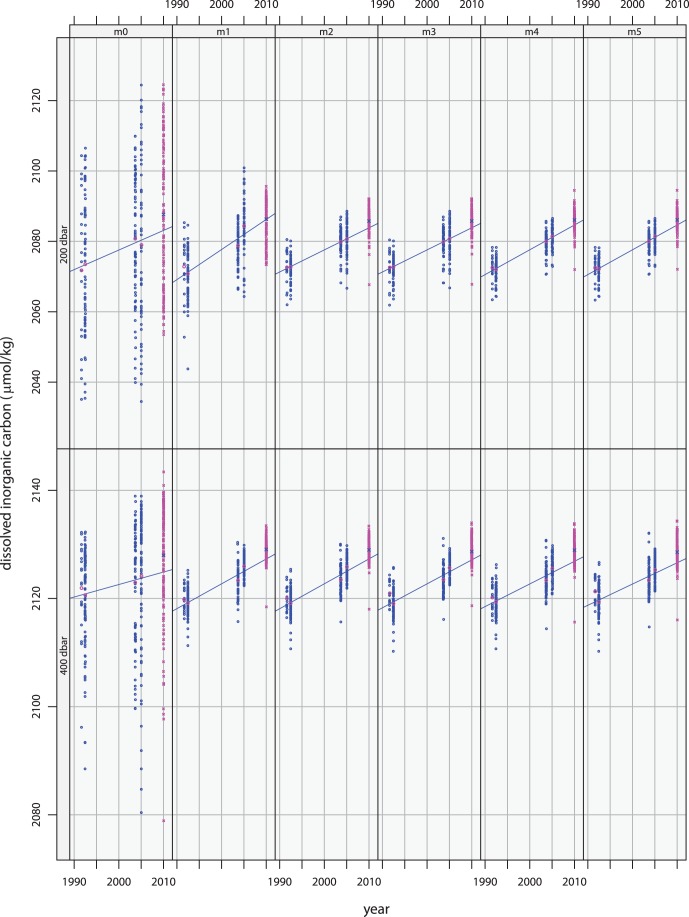
Projections based on the first four surveys. Blue circles indicate data from the first four surveys with environmental variability removed using the model indicated in the panel label. The regression lines have been fitted to these data. Magenta crosses indicate data from the most recent survey. Overlays in contrasting colors indicate the means of the filtered data for each of the five surveys.

Figure 4 shows the filtered data from these surveys as blue circles. Because the means have been restored, the panels for the default models, m0 at 200 and 400 dbar, show the actual measured values for dissolved inorganic carbon. The other panels show the data after they have been adjusted by the indicated model to remove natural variability. Note that, for all of the non-default models in both layers, the spread of the adjusted values is considerably less than that of the measured values, indicating that each of these models does indeed explain a sizable fraction of the variance. The filtered data in each panel are somewhat different, as each model provides a slightly different view of non-anthropogenic variability. With much better sampling, more regressors might safely be used and a better view of the natural variability might be possible. But given the available data, differences between models provide an indication of the uncertainties associated with the filtering.

Because of the limited nature of the sampling provided by the repeated surveys, overfitting is a concern. Using too many regressors to account for carbon’s environmental co-variability can limit a models ability to filter new data. Additional regressors can confuse the idiosyncrasies of the earlier data with meaningful information, causing the model to expect those same idiosyncrasies to manifest in data from the most recent survey. Thus, models m3, m4, and especially m5 are more likely to be overfitting the data than the other models.

To indicate the rate at which anthropogenic carbon has been accumulating during the temporal span of the first four surveys, a linear function of time has been fitted to the filtered values, as is illustrated in figure 4. The accumulation rates given by the slopes of the regression lines are listed in Table 3 for each model. Note that all methods for modeling natural variability yield higher accumulation rates than the those provided by m0. Also, the estimated rates indicate faster accumulation in the 200 dbar layer than in the 400 dbar layer. As these rate estimates were determined from the data from the first four surveys, they should be associated with the mean date of those observations – 1999.524 in the 200 dbar layer and 1999.424 in the 400 dbar layer. (To compute the fraction of year corresponding to month and day, observational dates were first converted to Julian days.).

**Table 3:** Accumulation rates inferred from data from first 4 surveys (1992–2005).

	200 dbar	400 dbar
m0	0.557 (0.235)	0.231 (0.111)
m1	0.855 (0.081)	0.459 (0.025)
m2	0.628 (0.045)	0.461 (0.024)
m3	0.628 (0.045)	0.443 (0.024)
m4	0.705 (0.036)	0.418 (0.026)
m5	0.705 (0.036)	0.393 (0.027)

Units are *µ*mol/kg/yr. Standard errors are indicated in parentheses.

Since each model characterizes the natural environmental variability differently, model-to-model differences in accumulation rate should be regarded as an indication of the uncertainty of the rate estimates stemming from the uncertainty of the environmental variability. Fitting the models to one pair of surveys and predicting for the other pair provided a quantitative measure of the accuracies of the rate estimates in an earlier study [1]. Here, a similar measure is provided by the ability to predict the anthropogenic component of the carbon measured in the most recent survey using models fitted to the four earlier surveys.

## Predictions

After being fitted to the data from four earlier surveys, the models of Table 2 can be used with the values for their regressors measured during the most recent survey to remove the environmental variability from the new inorganic carbon data. These adjusted values are shown along with those from the earlier surveys in figure 4. Note that the regression line, which was fitted to the filtered data from first four surveys, does pass close to the center of the filtered data from the most recent survey. This indicates that, whichever model of environmental co-variability is used to account for natural variability, the following temporal regression does a fair job at projecting the rate at which carbon has been accumulating.

But each regression line passes below the blue cross marking the mean of the adjusted data. In other words, there is a consensus among models in both layers that more carbon has accumulated than should be expected based on the rates estimated from the earlier surveys. The amount that it exceeds expectations depends on the model used to account for natural environmental variability. Table 4 list the mean differences between the data filtered by the models and corresponding values predicted by the regression lines. This table also lists the errors associated with those differences, which include the standard error of the mean differences and the standard error of the temporal prediction at the mean date of the survey combined as the square root of the sum of the squares.

**Table 4:** Mean differences between the anthropogenic component of the 2009–2010 data and their counterparts predicted by extrapolating the accumulation rates inferred from the first 4 surveys.

	200 dbar	400 dbar
m0	4.52 (3.40)	3.09 (1.59)
m1	0.07 (1.13)	1.76 (0.34)
m2	1.90 (0.64)	1.67(0.33)
m3	1.92 (0.64)	1.55 (0.34)
m4	1.43 (0.51)	2.05 (0.37)
m5	1.44 (0.51)	1.96 (0.39)

Units are *µ*mol/kg. Errors indicated in parentheses include both the standard error of the mean distance from the regression line and the standard error of the regression line at the mean date of the survey.

The largest excess in both layers is for the default models where nothing is done to account for natural variability. On the other hand, data for the 200 dbar layer that have been filtered by model m1, which uses co-variability of carbon with potential density to characterize natural variability, are quite well centered on the regression line, and the tabulated error indicates that the extrapolated accumulation rate can be considered a perfect prediction. In contrast, model m2, which uses co-variability with both nitrate and potential density, leads to an underestimation of 1.90 *µ*mol/kg of accumulated anthropogenic carbon; as the accumulation rate in the upper layer resulting from m2 filtering was estimated to be 0.628 *µ*mol/kg/yr, the excess accumulation is equivalent to what would have been expected three years later. This excess accumulation is three times larger than the error of 0.64 *µ*mol/kg. Similarly, except for m2 in the 200 dbar layer, the excess accumulation for all filtering models is greater than the tabulated error. The fact that nine of the ten models clearly underestimate the anthropogenic signal in the new data suggests that the accumulation rates in both layers might be increasing.

To gauge the size of the excess accumulation of anthropogenic carbon, the distances of the blue crosses from the regression lines can be compared with the similar offsets of the magenta circles marking the means of the data from the previous surveys. While the excess accumulation is generally larger, it can nevertheless be regarded as being of similar magnitude and as providing support for the accumulation rate being constant. The difficulty in deciding whether the rate of accumulation actually has been increasing or whether it simply should be revised upward in light of the new observations is clearly due to the paucity of the available observations.

## Revised Analyses Using All Five Surveys

**Figure 5:**
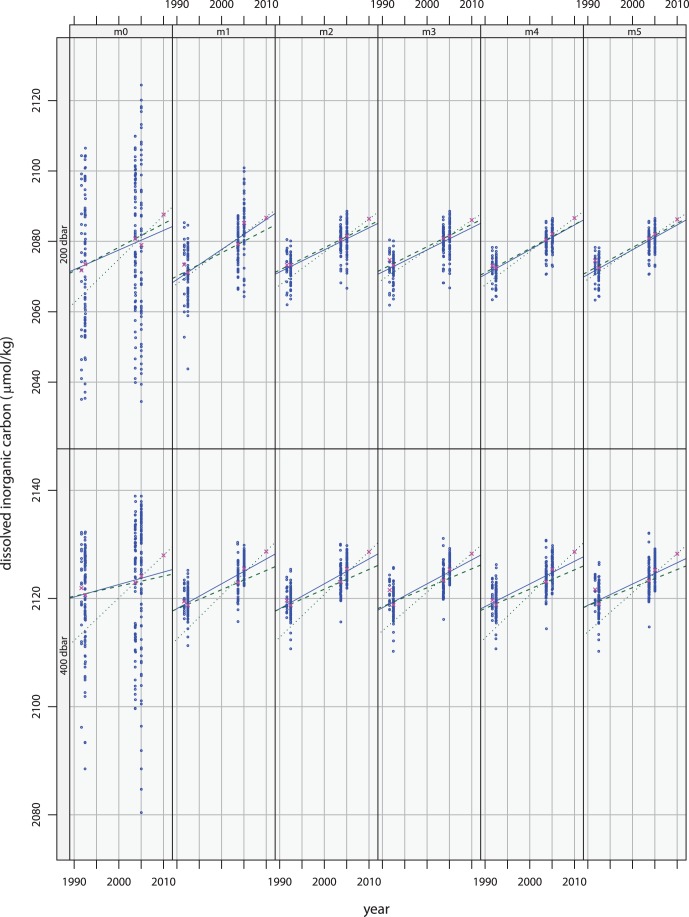
Analysis of data from all five surveys. Environmental variability has been removed using the model indicated in the panel label. Filtered data are indicated in blue. Magenta indicates the means of the filtered data for each survey. Solid black lines indicates fits to all 5 surveys, green dashed lines, fits to surveys 1–3, and green dotted lines, fits to surveys 3–5.

The most recent survey provides additional information about the nature of the environmental variability as well as about accumulated carbon from anthropogenic CO_2_, so it is useful to redo the analysis using data from all five surveys. Figure 5 illustrates the results. Qualitatively each panel looks quite similar to its counterpart in figure 4. Even the groupings of points are similar. A close look reveals that the blue regression lines, which have been fitted to data from all five surveys are much closer to the fifth-survey means than are their counterparts in figure 4. Clearly, the points have moved and the slopes of the regression lines are different.

Table 5 lists the revised rates. Note that for each of the models in each of the layers the revised rates are larger than those based on only the first four surveys, which are listed in Table 3, but the amounts of increase are not the same for all models. In particular, using silicate to help to characterize environmental variability in the upper layer now allows the m3 rate to be distinguished from m2 rate and the m5 rate from the m4 rate. Also note that the standard errors are smaller than for the previous estimates of the accumulation rates due to two factors: the increase in the number of samples and the increase in the interval over which the slope has been determined. The fact that the various models yield different results for the rates illustrates the difficulty in accounting for natural environmental variability. Consequently, model to model differences in the rates should be regarded as a measure of uncertainty. In the 200 dbar layer the accumulation rate is likely to be in the range of 0.7–0.8 *µ*mol/kg/yr, and in the 400 dbar layer, 0.5–0.55 *µ*mol/km/yr.

**Table 5:** Accumulation rates inferred from data from all 5 surveys (1992–2010).

	200 dbar	400 dbar
m0	0.780 (0.162)	0.386 (0.075)
m1	0.864 (0.052)	0.547 (0.016)
m2	0.728 (0.030)	0.543 (0.016)
m3	0.695 (0.032)	0.502 (0.017)
m4	0.778 (0.024)	0.537 (0.016)
m5	0.745 (0.026)	0.492 (0.018)

Units are *µ*mol/kg/yr. Standard errors are indicated in parentheses.

## Split Interval Analyses

After accounting for natural variability seen in the data from all five surveys, it is possible to check whether or not anthropogenic carbon has been accumulating at a constant rate over the two decades by separately fitting trend lines to filtered data from the first three surveys and from the last three surveys. In doing so, the data from the 2003 P06 survey contribute to both fits. The results are indicated in figure 5 by the green lines, where dashes correspond to fits to the 1st, 2nd, and 3rd clusters of points and dots to fits to the 3rd, 4th, and 5th. In all panels the slopes determined from the more recent surveys can be seen to be larger than their counterparts from the earlier surveys.

The slopes of these regression lines, which provide estimates of the accumulation rates for the two periods, are listed in Table 6. All estimates of accumulation rates clearly increase from the first period to the second. The standard errors are all larger than those of Tables 3 and 5 due to the fits being determined by fewer data and over shorter intervals. Nevertheless, the differences between the earlier and later estimates appear appreciable when compared to the sizes of the errors. T-tests show all increases in accumulation rates to be significant at the 95% level or higher, except for the case with no adjustment for background variability in 200 dbar layer. Similarly, while the exceptionally low 1991–2003 rate for the 400 dbar m0 case leads to an extremely high level of significance for the increase, it might be better viewed as an indication of a problem associated with ignoring carbon’s natural variability.

**Table 6:** Accumulation rates before and after the 3rd survey.

	200 dbar		400 dbar	
	1991–2003	2003–2010	1991–2003	2003–2010
m0	0.67 (0.26)	1.24 (0.45)	0.19 (0.13)	0.79 (0.22)
m1	0.66 (0.08)	0.95 (0.14)	0.36 (0.03)	0.81 (0.04)
m2	0.63 (0.05)	0.94 (0.09)	0.37 (0.03)	0.79 (0.04)
m3	0.64 (0.05)	0.84 (0.09)	0.35 (0.04)	0.72 (0.05)
m4	0.68 (0.04)	0.95 (0.07)	0.36 (0.03)	0.80 (0.04)
m5	0.69 (0.05)	0.85 (0.07)	0.33 (0.04)	0.72 (0.05)

Adjustments for non-anthropogenic variability is based on data from all 5 surveys. Units are *µ*mol/kg/yr. Standard errors are indicated in parentheses. Note that all treatments of natural variability indicate accelerating rates of accumulation in both layers.

## Discussion

In the upper ocean in the neighborhood of 32.5°S and 150°W an anthropogenic signal of increasing dissolved inorganic carbon is clearly seen. This is true whether or not any attempt is made to account for the effects of natural variability through carbon’s co-variability with other environmental observables. Exploiting this co-variability leads to a quantitative indication of the uncertainty of the accumulation rate.

When added to the earlier data, those from the most recent survey provide upwardly revised accumulation rates. The nearly linear alignment of the means from the five surveys suggests that the accumulation rate has been nearly steady for two decades. However the fact that rates based on the first four surveys underestimate the anthropogenic component of carbon measured during the fifth survey suggests that the rate has been increasing. This conclusion is also supported by the split interval analyses, which show a clear increase in rates. However, the uncertainty in rates stemming from our limited ability to characterize the natural variability is of a similar magnitude as the increase in rates, so this conclusion should be taken with caution.

These results apply only to this small region in the southeast Pacific. It would be interesting to see the results for other regions where multiple surveys provide sampling that is comparable to or better than what was available for this study. Further consensus of an accelerating accumulation in other regions would reinforce the notion that anthropogenic carbon’s accumulation rate is increasing.
